# Hypermethylation of Synphilin-1, Alpha-Synuclein-Interacting Protein (SNCAIP) Gene in the Cerebral Cortex of Patients with Sporadic Parkinson’s Disease

**DOI:** 10.3390/brainsci7070074

**Published:** 2017-06-27

**Authors:** Khashayar Dashtipour, Ali Tafreshi, Charles Adler, Thomas Beach, Xin Chen, Geidy Serrano, Stephanie Tashiro, Charles Wang

**Affiliations:** 1 Department of Neurology/Movement Disorders, School of Medicine, Faculty of Medical Offices, 11370 Anderson, Suite B-100, Loma Linda, CA 92354, USA; tafreshi@usc.edu (A.T.); stashiro@llu.edu (S.T.); 2 School of Pharmacy, Loma Linda University, Loma Linda, CA 92354, USA; 3 Center for Genomics & Department of Basic Sciences, School of Medicine, Loma Linda University, Loma Linda, CA 92354, USA; xchen@llu.edu (X.C.); chwang@llu.edu (C.W.); 4 Department of Neurology, Mayo Clinic College of Medicine, Scottsdale, AZ 85255, USA; cadler@mayo.edu; 5 Banner Sun Health Research Institute, Sun City, AZ 85351, USA; Thomas.Beach@bannerhealth.com (T.B.); Geidy.Serrano@bannerhealth.com (G.S.)

**Keywords:** epigenetics, DNA methylation, Parkinson’s disease, genetics, SNCAIP, synphilin-1

## Abstract

Objective: To determine and compare DNA methylation patterns between patients with Parkinson’s disease (PD) and age- and sex-similar matched non-PD controls. Background: Epigenetic regulation is one of the major mechanisms for an organism to respond to the environment through changes in gene expression and has been implicated in numerous disease processes. We would like to examine epigenetic modification patterns that may predispose or protect against PD. Methods: Frozen tissue samples of the human cerebral cortex from 12 PD patients and 12 subjects without PD pathology were obtained. Genome-wide DNA methylation profiling was performed using the Illumina HumanMethylation450 BeadChip array. Differential methylation was defined as a mean methylation level difference (delta β) of at least 0.20 (Δβ ≥ 0.20). Methylation regions with an absolute delta β value ≥ 0.20 were selected for further gene function studies. Results: We identified 2795 differentially methylated CpG sites in the frontal cortex of PD cases with a detection *p*-value of ≤ 0.01 and 328 differentially methylated CpG sites with a detection *p*-value of ≤ 0.001. A pattern of robust hypermethylation of synphilin-1, α-synuclein-interacting protein (SNCAIP) gene was found in the brain of PD cases (*p* = 4.93 × 10^−7^ and delta β = 0.60). Conclusion: Our findings support a link between SNCAIP methylation and PD risk. Hypomethylation of SNCAIP may function to protect against PD. The current results may suggest that the methylation status of SNCAIP could be useful as a marker in PD diagnosis and treatment and warrants further investigation.

## 1. Introduction

The etiopathogenesis of neurodegenerative disorders including Parkinson’s disease (PD) is not clear but interactions between genes and environmental pathogens seem to play a major role in PD [1]. Multiple genes have been discovered that contribute to the development of PD but still the majority of the sporadic PD patients do not carry a specific identified gene [2,3]. On the other hand, multiple environmental factors have been suggested to produce oxidative stress, inflammation, and cell loss in localized brain regions, leading to PD [4]. Interestingly, the majority of our population is still exposed to these suggested environmental factors such as pesticides, heavy metals, trauma, agent orange, etc., but never develop PD [5]. Thus, gene–environment interactions seem to play a major role in the initiation of the PD disease process. Another possibility would be that there exist certain ubiquitous environmental factors that can cause PD in all people, but that the majority of the population carries one, or most likely multiple, unknown genes that protect against the development of the disease [5].

Epigenetics, particularly DNA methylation, is a potential mechanism whereby the environment affects the genome [6]. There is a growing body of evidence demonstrating the associations between chronic progressive diseases and aberrant DNA methylation modifications [7]. Epigenetic mechanisms are essential for development and differentiation and allowing an organism to respond to the environment through changes in gene expression patterns. We believe there would be great value in identifying epigenetic alternations and the patterns that predispose or protect against PD. These changes accumulate over time in response to lifestyle and environmental exposures and subsequently affect gene regulation and disease susceptibility.

Altered DNA methylation in the brain has been observed in many neurodegenerative and neurological disorders [7]. Several studies have found aberrant methylation of several genes related to the pathogenesis of PD [8]. These studies showed that decreased methylation of CpG islands at intron 1 of the SNCA gene (synuclein, α) resulted in increased α-synuclein gene expression [9]. A genome-wide DNA methylation study also revealed global hypomethylation in the brains of PD patients [10]. Similarly, a recent study investigating the genome-wide DNA methylation profiles of brain and blood samples of PD patients and control subjects revealed a set of genes with differential methylation (DM) in brain and blood, with most of these loci previously reported in pathogenesis of PD [11]. In this study, the authors identified 2908 CpG with DM in the brain and 3897 CpG in the blood of PD cases. Using DM analysis, they further identified 317 probes in brain and 476 in blood with increased methylation, and 2591 probes in brain and 3421 in blood with decreased methylation in patients with PD. Subsequently, after refining the analysis to the fraction of autosomal probes, they showed differential methylation in a total of 174 genes in the brain (84 with increased and 90 with decreased methylation in PD) and 233 genes in blood (127 with increased and 106 with decreased methylation in PD). Finally, using the unsupervised hierarchical clustering of the individual methylation profiles detected from blood, they demonstrated a clear separation between control and PD cases [11].

Considering the possible interaction between environment and organism, we conducted a genome-wide DNA methylation analysis in brain tissue from 24 brain autopsies to provide preliminary information regarding the methylation pattern of patients with PD. In this study, we compared 12 PD subjects’ DNA methylome to that of 12 sex- and age-matched control subjects, using Illumina Infinium HumanMethylation 450 BeadChip which interrogates more than 450,000 methylation sites across the human genome.

## 2. Methods

**Sample collections:** The frozen tissue samples of the human cerebral cortex (superior frontal gyrus at the level of genu of the corpus callosum) from 12 PD patients and 12 subjects without PD pathology or other neurodegenerative disorders (age- and sex-similar matched controls) were obtained from the Banner Sun Health Research Institute Brain and Body Donation Program [12].

**DNA methylome analysis:** A genome-wide DNA methylation profiling was performed using the Illumina Infinium HumanMethylation 450 BeadChip Kit at the University of California, Los Angeles Neuroscience Genomics Core. The DNA microarray was carried using 500 ng genomics DNA which was treated with the EZ DNA Methylation-Gold Kit (Zymo Research, Orange, CA, USA) according to the manufacturer’s protocol. Methylation fraction values for the autosomal chromosomes were performed using the Infinium HumanMethylation 450 BeadChip assay [13]. The intensity of the methylated probe relative to the total probe intensity for each site represented the fractional level of methylation at that site in the sample. These values were adjusted for internal controls with Illumina’s GenomeStudio software (Illumina, Incorporated, San Diego, CA, USA).

Bisulfite treatment, whole genome amplification, labeling, hybridization and scanning were performed following the manufacturer’s protocols for the EZ DNA Methylation-Gold and Infinium HumanMethylation 450 BeadChip kits. The methylation data generated by the array were analyzed using the Illumina GenomeStudio software package. The methylation status of a specific CpG site was expressed as β values, calculated as the ratio of the fluorescence intensity signals of the methylated (M) and unmethylated (U) alleles, β = Max(M,0)/[Max(M,0) + Max(U,0) + 100]. β values range between 0 (non-methylated) and 1 (completely methylated).

**Bioinformatics data analysis:** Bioinformatics data mining was carried out in the Loma Linda University Center for Genomics (Loma Linda University, Loma Linda, CA, USA). Illumina data array was processed using Partek Genomics Suite, under the assumption that β-values are normally distributed. In each individual, probes with a detection *p* value > 0.01 were removed from later analyses. We excluded CpG sites localized on sex chromosomes to prevent variability among samples within a tissue set related to gender. Differential methylation analysis between sets of samples representing two tissues was performed by using Bioconductor package minfi. Significant differentially methylated regions (DMRs) were selected with *p*-value < 0.01. DMRs where methylation values for PD were less than controls for the majority of individual CpGs were considered hypomethylated; otherwise, they were annotated as hypermethylated. Hierarchical clustering and principal component analysis were performed for a significant DMR list by using Partek Genomics Suite 6.0.

The autosomal probe sequences were annotated by using Illumina HumanMethylation 450 K BeadChip annotation file. We defined promoter regions as ±2 kb from transcriptional start sites (TSS), gene body regions as transcription start to transcription end after excluding the first 2 kb of the gene, and intergenic regions as those not annotated by the preceding categories. We also annotated individual CpG in context with CpG island (using Bioconductor package IlluminaHumanMethylation450k.db), CpG shore (±2 kb of island), CpG shelf (±2 kb of shore) and CpG sea (regions outside the previous three categories).

**Gene ontology analysis and data set signature comparison:** To identify enriched gene functions associated with the DMRs in PD brain tissue samples, we performed gene ontology (GO) analysis by using Partek Genomics Suite 6.0.

At the end, the methylation regions with an absolute delta β value ≥ 0.20 were selected for further gene function studies.

## 3. Results

### 3.1. Demographics

Twenty-four samples from the cortex of subjects were compared from two groups of PD and non-PD subjects. Table 1 shows the demographics for each group. Mean age of each group was 78.9 and 79.4 years old for PD and control respectively. PD patients were all male however there were three females in the control group. Unified Parkinson disease rating scale (UPDRS) was measured during ON or OFF condition or both. UPDRS was available for eleven off of twelve PD subjects. Using the Unified Staging System for Lewy Body Disorders, seven patients were stage III and five were stage IV (Table 1).

### 3.2. DNA Methylation Analysis

**Figure 1:**
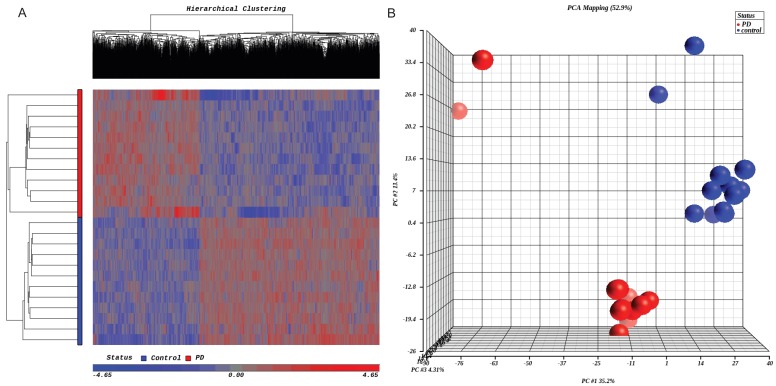
Hierarchical clustering analysis (**A**) and principal component analysis (**B**) based on differentially methylated CpGs between PD and controls.

We identified 2794 differentially methylated CpG sites in the frontal cortex of PD cases with a detection *p*-value of ≤ 0.01 and 328 differentially methylated CpG sites with a detection *p*-value of ≤ 0.001. The majority of differentially methylated regions were hypomethylated, suggesting overall hypomethylation in the brain of PD cases compared to controls (Table 2). The observed differences in DNA methylation between groups were small but widespread. A pattern of robust hypermethylation of synphilin-1, α-synuclein-interacting protein (SNCAIP) gene was found in the brain of PD cases (*p* = 4.93 × 10^−7^ and delta β = 0.60). Unsupervised hierarchical clustering of samples based on genome-wide DNA methylation profiles clearly delineated PD cases from controls (Figure 1).

## 4. Discussion

PD is a synucleinopathy caused by an accumulation of misfolded α-synuclein. Lewy bodies, which are the pathological hallmark of PD, contain an abundant amount of this protein. While α-synuclein plays a role in the pathogenesis of Parkinson’s and other neurological diseases, it also has important physiological functions in normal neural tissue.

### 4.1. Physiological Role of α-Synuclein

α-synuclein (encoded by the gene SNCA) is a natively unfolded protein comprised of 140 amino acids, predominantly localized in the presynaptic terminals of neurons. α-synuclein is abundantly expressed in the human brain, constituting as much as 1% of protein content in the cytosol and is present at high levels in the neocortex, hippocampus, substantia nigra, thalamus and cerebellum [14]. Considered an unstructured soluble protein, un-mutated α-synuclein may occur physiologically as a monomer or a helically folded tetramer that resists aggregation [15].

Several studies have suggested that α-synuclein regulates levels of phosphatidic acid in neurons. Specifically, α-synuclein is thought to inhibit the function of phospholipase D2, which synthesizes phosphatidic acid [16]. Researchers believe that α-synuclein therefore participates in important feedback loops for phosphatidic acid synthesis, preventing its levels from becoming too high [17]. Phosphatidic acid is widely recognized as an important molecule for the budding of intracellular vesicles [18]. Thus, the physiological function of α-synuclein is thought to be the regulation of phosphatidic acid and vesicle budding, which is important in neuronal function.

α-synuclein has been shown to play an important role in the formation of SNARE complexes. These complexes are required for the fusion of synaptic vesicles with the presynaptic membrane, making α-synuclein doubly important in cell signaling. Specifically, Burré and colleagues [19] infected neurons with a lentivirus that expressed α-synuclein. They found that the addition of this lentivirus linearly increased the formation of SNARE protein complexes in these neurons. This led researchers to conclude that α-synuclein plays the physiological role of supporting SNARE complex assembly in nerve cells.

Lastly, α-synuclein has been implicated in microtubule formation [20]. Alim and colleagues [20] found that, at various concentrations, a solution of tubulin polymerized effectively in the presence of α-synuclein, but not mutated α-synuclein. This led the researchers to conclude that α-synuclein is important for the polymerization of tubulin particles into microtubules. Microtubules have long been implicated in the vital process of axoplasmal transport [21]. Thus, α-synuclein is likely to be very important in physiological nerve cell function.

### 4.2. Physiological Role of Synphilin-1

Another protein associated with the neurodegeneration of Parkinson’s disease is synphilin-1.

Synphilin-1 has been shown to interact with the protein tbp7, which is part of the cellular proteasome. Specifically, Marx and colleagues [22] mixed a solution of tagged synphilin-1 with tbp7 protein (protein S6 ATPase) to observe any binding interaction between the proteins. They found that synphilin-1 precipitated from solution along with a subunit of the tbp7 protein, leading the researchers to conclude that synphillin-1 binds to the tbp7 protein in cells. Accordingly, this result indicates that synphilin-1 may play a key role in the cellular proteasome, which is important in ubiquitination and protein degradation [23], a vital process in cell function.

Another study found that synphilin-1 is localized with lipid fractions in brain samples of rats [24]. Specifically, Murray and colleagues [24] tagged rat brain extracts with anti-synphilin-1 antibodies and conducted immunohistochemistry to locate synphilin-1 protein in lipid fractions. This finding led some researchers to conclude that synphilin-1 participates in synaptic vesicle formation and secretion [25].

Furthermore, using immunocytochemistry and in silico rendering, other researchers have demonstrated that synphilin-1 also interacts with phosphoprotein phosphatase 1 [26]. The authors hypothesize that synphilin-1 may localize phosphoprotein phosphatase 1 to the synaptic membrane and modulate vesicle release, although this is mostly speculation. Other researchers have described that this phosphatase plays important roles in maintaining long-term potentiation/signal strength or modulating axonal ion channel opening and closing [27,28].

### 4.3. Physiological Interaction between α-Synuclein and Synphilin-1

The interaction between these two proteins is primarily associated with the pathophysiology of Parkinson’s disease. However, these two proteins have been shown to interact under physiological conditions, not in the disease state. Specifically, Ribeiro and colleagues [29] used immunoprecipitation and chromagen staining to find that α-synuclein and synphilin-1 localize together in presynaptic axon terminals. Researchers speculate that synphilin-1 promotes α-synuclein binding to synaptic vesicle membranes [25]. In vitro studies showed that overexpression of Synphilin-1 inhibits the degradation of α-synuclein by the 20S proteasome, due in part to the interaction of the Synphilin-1 central region 331–355 with the N-terminal region 1–60 of α-synuclein [30]. The overexpression of synphilin-1 by cell transfection increases the half-life of α-synuclein, resulting in an increased turnover [30]. Furthermore, Synphilin-1 is present in Lewy bodies, interacting with α-synuclein in vivo and in vitro and promotes its sequestration into aggresomes [31].

### 4.4. Hypermethylation of Synphilin-1

Our data shows a robust hypermethylation of the SNCAIP gene in the brain cortex of PD case, suggesting that this gene is down-regulated in PD subjects. It seems that this protein interacts with α-synuclein and our data supports the previous in vivo studies, suggesting the protective effect of SNCAIP. Accordingly, we hypothesize that SNCAIP might serve a protective role in the development of PD in normal patients without down-regulated SNCAIP.

The small sample size of this study is one of the limitations of our analysis. Looking at the methylome pattern of larger samples with minimizing environmental variables such as medical therapies is warranted. Furthermore, there was at high rate of co-occurrence of dementia with PD in our samples. Thus, given the nature of this study, it is difficult to comment on any possible interactive role that dementia may play in our findings. It is possible that the presence of dementia can have epigenetic effects; likewise, it is also possible that the epigenetic modifications present may be particularly related to PD with dementia, and not PD alone. While this is not a particularly critical distinction to make in this study, given that dementia is one of the sequelae of PD, this may be an important avenue for further research: it is possible that different epigenetic modification patterns tend to be present in patients who have certain motor or non-motor complications (i.e., freezing of gait, dyskinesia, depression, cognitive impairment, etc.) versus those who do not. Broadly, it may prove fruitful to examine the role that epigenetics may play in the appearance of specific components or subtypes of neurodegenerative disease

## Figures and Tables

**Table 1:** Demographics.

Case	Age	Gender	Motor UPDRS ON	Motor UPDRS OFF	LB Stage	Pathology and Diagnostic Summary
PD1	69	M		21	3	History of PD and dementia; Status post SN DBS; Old microscopic infarcts
PD2	75	M	32		3	History of PD and dementia; Status post SN DBS; Alzheimer type II astrocytosis
PD3	81	M		71	3	History of PD and dementia; Cerebral AA
PD4	78	M	51		3	History of PD and dementia; Old cortical microscopic infarct
PD5	80	M		40	3	History of PD and dementia; Status post SN DBS
PD6	85	M		59	4	History of PD and dementia; Cerebral WM rarefaction; Microscopic changes of AD, insufficient for diagnosis
PD7	83	M		37	4	History of PD and dementia; Cerebral WM rarefaction; Microscopic changes of AD, insufficient for diagnosis
PD8	86	M	68	76	4	History of PD and dementia
PD9	69	M		45	4	History of PD and dementia; Microscopic changes of AD, insufficient for diagnosis
PD10	76	M	31		3	History of PD and dementia; NT
PD11	81	M			3	History of PD and dementia; Cerebral WM rarefaction
PD12	84	M	26		4	History of PD and dementia; Cerebral WM rarefaction
C1	81	M	NA	6.5	0	microscopic changes of early or mild AD but insufficient for diagnosis
C2	86	M	NA	17.5	0	brain showing only non-specific changes
C3	95	F	NA		0	Unruptured saccular aneurysm ACA; History suggestive of RLS
C4	83	F	NA	13	0	Glial and neuronal tauopathy; Cerebral WM rarefaction
C5	82	M	NA	0	0	Microscopic changes of AD, insufficient for diagnosis; Old microscopic infarction
C6	88	F	NA		0	Microscopic changes of AD, insufficient for diagnosis; Small old hemorrhagic lacunar infarct, Cerebral WM rarefaction
C7	73	M	NA	4	0	Microscopic changes of AD, insufficient for diagnosis
C8	76	M	NA	3	0	Brain normal for age
C9	80	M	NA		0	Brain normal for age; Microscopic features of AD, insufficient for diagnosis
C10	61	M	NA		0	Brain normal for age; Microscopic features of AD, insufficient for diagnosis
C11	69	M	NA		0	very mild microscopic changes of AD, non-diagnostic
C12	79	M	NA	3	0	Normal aging changes; Cerebral WM rarefaction; Old microscopic infarct

AA: Amyloid angiopathy; PD: Parkinson’s disease; C: Control; SN: Substantia Nigra; DBS: Deep Brain Stimulation; NT: Neurofibrillary tangles; PUT: Putamen; AD: Alzheimer’s disease; WM: White matter; BG: Basal ganglia; ACA: Anterior cerebral artery; LRS: Restless leg syndrome.

**Table 2:** Differentially methylated CpGs.

Cutoff Criteria	12 PD vs. 12 C		
*p*-value	Hypo	Hyper	Total
<0.01	1755	1039	2794
